# Supplementary material for: Stable isotope evidence of anthropocene disruption in African softshell turtle foraging
Source: PLoS One. 2026 Feb 11;21(2):e0339589. doi: 10.1371/journal.pone.0339589 (PMC12893573; doi:10.1371/journal.pone.0339589)
Supplement: S1 File — (DOCX) [file pone.0339589.s001.docx]

**S1 Text. Supplementary Information**

*Archaeological Sites*

**Tell-Fadous-Kfarabida**

Tell-Fadous-Kfarabida, situated along the central Levantine coast in present-day Lebanon, is an Early Bronze Age site that has produced the oldest African Softshell Turtle (*Trionyx triunguis*) archaeological specimen analysed in this study. These remains, dated through radiocarbon analysis to approximately 5,000–4,500 years ago [(Höflmayer et al. 2014)](https://paperpile.com/c/2eYXze/Fbxo) , provide insight into the site's environmental and subsistence practices. The coastal settlement had access to both marine and brackish-water resources (although no perennial river); with evidence that shellfish collection and marine exploitation playing a notable role [(Genz et al. 2016)](https://paperpile.com/c/2eYXze/vu3N). The presence of *T. triunguis* remains, though limited, suggests occasional use of freshwater turtles, probably for food, within the broader scope of Bronze Age resource utilisation.

**Tell el-Burak**

Tell el-Burak, also located in the central Levant, has yielded three *T. triunguis* specimens analysed in this study, all attributed to Iron Age II phases A, B, and C, dating to approximately 2,600–2,400 years ago [(Vermeersch et al. 2022)](https://paperpile.com/c/2eYXze/NfKj). While *T. triunguis* remains are relatively scarce in the assemblage, their presence suggests occasional interaction with freshwater species, which is further supported by the presence of freshwater fish [(Winter 2023)](https://paperpile.com/c/2eYXze/x1oQ).

The turtle assemblages at both sites are highly fragmented, consisting of skeletal elements that often preclude definitive osteological species identification. This fragmentation may be due to post depositional processes, or specific human activities related to the use and disposal of *T. triunguis* remains. The limited number of remains suggests a lower degree of interaction with *T. triunguis* compared to sea turtles [(Çakırlar, Koolstra, and Ikram 2021; de Kock et al. 2023)](https://paperpile.com/c/2eYXze/R0hj+C9rk), potentially reflecting differences in subsistence strategies, ecological availability, ease of capture or cultural practices. Nevertheless, their presence at these Levant sites indicates that *T. triunguis* was at least occasionally utilised by ancient communities in this region, and contributes to our understanding of human-animal interactions and freshwater resource exploitation in Bronze and Iron Age societies.

*Archaeological Sample preparation*

*i) ZooMS*

Archaeological bone specimens underwent initial processing involving surface scraping using a rotary tool, followed by analysis conducted within a keratin-exclusion facility situated at the Globe Institute of the University of Copenhagen. Protein extraction from the bone specimens, encompassing both ancient and a museum reference sample, adhered to a methodology akin to that delineated by Buckley et al. [(Buckley et al. 2009)](https://paperpile.com/c/2eYXze/s7q5o). Briefly, 10 milligrams of bone material underwent demineralization through immersion in 1.2 M hydrochloric acid (HCl) and subsequent placement on a refrigerated rotary apparatus set at 4°C until complete demineralization took place. The ensuing step involved protein extraction utilising a guanidine hydrochloride (GuHCl) extraction buffer, followed by quantification employing a bicinchoninic acid (BCA) assay. Subsequently, enzymatic digestion was achieved through the addition of trypsin. Conclusively, peptide desalting was executed by ZIPtip using Pierce™ C18 Spin Tips.

Samples were then precisely spotted onto a 384-spot Matrix-Assisted Laser Desorption/Ionization Time-of-Flight (MALDI-TOF) plate and subsequently subjected to MALDI-TOF spectrometric analysis, conducted at the Bioarchaeology facilities of the University of Cambridge.

In addition, shotgun proteomic data was acquired from a museum reference specimen. This sample underwent analysis via nanoflow liquid chromatography utilising an EASY-nLC 1200 system, coupled to an Exploris 480 mass spectrometer (both Thermo Fisher Scientific). Detailed information regarding the mass spectrometry parameters employed in this analysis can be found in Winter et al. (2023).

LC–MS/MS spectra were compared against the complete COL1 sequences for the following species: *Chelonia mydas*, *Chelonoidis abingdonii*, *Chrysemys picta bellii*, *Dermochelys coriacea*, *Gopherus evgoodei*, *Mauremys reevesii*, *Pelodiscus sinensis*, *Terrapene carolina triunguis*, and *Trachemys scripta elegans*. These sequences were retrieved from NCBI and analyzed using the open search algorithm in the pFind software [(Wang et al. 2007)](https://paperpile.com/c/2eYXze/IJGAb). Amino acid substitutions identified through this initial search were subsequently verified or rejected with MaxQuant version 2.0.3.0 [(Cox and Mann 2008)](https://paperpile.com/c/2eYXze/Q7mei). We performed both specific and unspecific searches, with the maximum peptide length in unspecific searches set to 40 amino acids. Precursor and fragment mass tolerances were set to the default for orbitrap mass spectrometers. Peptides exhibiting significant coverage of Y and B ions, particularly at varying amino acid positions, were manually examined and kept if deemed reliable. MALDI spectra were analysed using mMass software [(Strohalm et al. 2010)](https://paperpile.com/c/2eYXze/ZDHiT) according to established processing criteria [(Janzen et al. 2021)](https://paperpile.com/c/2eYXze/ngoBi). Initially, we focused on detecting the published sea turtle ZooMS biomarkers [(Harvey et al. 2019)](https://paperpile.com/c/2eYXze/jT3UK) to identify peptide variations. However, additional biomarkers were later discovered using pBuild [(Wang et al. 2007)](https://paperpile.com/c/2eYXze/IJGAb), see Panel S1.

*i) Stable Isotope Analysis*

*Sample preparation:*

Collagen extraction procedures of the four archaeological samples was conducted at the BioArCh facilities at the University of York following a modified Longin method [(Longin 1971)](https://paperpile.com/c/2eYXze/ny8et). Briefly, bone samples weighing approximately 500 milligrams underwent demineralization through exposure to acid, followed by gelatinization and subsequent freeze-drying. Detailed protocols are provided in [(Alexander et al. 2015, de Kock et al. 2023)](https://paperpile.com/c/2eYXze/YWyMi).

*Stable Isotope Analysis:*

Stable Isotope Analysis of extracted collagen was conducted at the Scottish Universities Environmental Research Centre (SUERC), situated at the University of Glasgow. Stable carbon (δ^13^C), nitrogen (δ^15^N), and sulphur (δ^34^S) isotopic compositions were determined utilising a Delta V Advantage continuous-flow isotope ratio mass spectrometer, which was interfaced with an IsoLink elemental analyzer via a ConfloIV system (Thermo Scientific, Bremen). The methodology is further detailed in [(Sayle et al. 2019)](https://paperpile.com/c/2eYXze/rpsK4).

Samples underwent combustion in the presence of oxygen within a singular reactor containing tungstic oxide and copper wires, maintained at a temperature of 1020°C. This process resulted in the production of nitrogen gas (N2), carbon dioxide (CO2), and sulphur dioxide (SO2). A magnesium perchlorate trap was utilised to remove water generated during the combustion phase, while gas separation occurred within a gas chromatography (GC) column, heated to temperatures ranging between 70°C and 240°C. Helium served as the carrier gas throughout the procedure. Subsequently, the N2, CO2, and SO2 gases were introduced into the mass spectrometer via an open split configuration within the ConfloIV system and subjected to analysis against reference gases corresponding to each analyte.

The International Atomic Energy Agency (IAEA) reference materials USGS40 (L-glutamic acid, δ^13^CVPDB = –26.39 ± 0.04 ‰, δ^15^NAIR = –4.52 ± 0.06 ‰) and USGS41a (L-glutamic acid, δ^13^CVPDB = 36.55 ± 0.08 ‰, δ^15^NAIR = 47.55 ± 0.15 ‰) were used to normalise δ^13^C and δ^15^N values. Two in-house standards (GS2, δ^34^SVCTD = –10.28 ± 0.18 ‰ and GAS2, δ^34^SVCTD = 18.56 ± 0.10 ‰) that are calibrated to the International Atomic Energy Agency (IAEA) reference materials IAEA-S-2 (silver sulfide, δ^34^SVCTD = 22.62 ± 0.08 ‰) and IAEA-S-3 (silver sulfide, δ^34^SVCTD = –32.49 ± 0.08 ‰) were used to normalise δ^34^S values. Results are reported as per mil (‰) relative to the internationally accepted standards VPDB, AIR and VCDT. Normalisation was checked using the marine collagen USGS88 (δ^13^CVPDB = –16.06 ± 0.07 ‰, δ^15^NAIR = 14.96 ± 0.14 ‰, and δ^34^SVCTD = 17.10 ± 0.44 ‰) and the well characterised Elemental Microanalysis IRMS fish gelatin standard B2215 (δ^13^CVPDB = –22.92 ± 0.10 ‰, δ^15^NAIR = 4.26 ± 0.12 ‰, and δ^34^SVCTD = 1.21 ± 0.24 ‰), which gave the values:

| ***USGS88*** ***Run Date: 07/10/2021***   - *δ¹³C₍VPDB₎: –16.25 ± 0.01 ‰ (n=5)* - *δ¹⁵N₍AIR₎: 15.05 ± 0.12 ‰ (n=5)* - *δ³⁴S₍VCTD₎: 17.36 ± 0.27 ‰ (n=5)*   ***Run Date: 18/10/2021***   - *δ¹³C₍VPDB₎: –16.27 ± 0.04 ‰ (n=3)* - *δ¹⁵N₍AIR₎: 14.82 ± 0.10 ‰ (n=3)* - *δ³⁴S₍VCTD₎: 17.35 ± 0.39 ‰ (n=3)*   ***Run Date: 21/10/2021***   - *δ¹³C₍VPDB₎: –16.29 ± 0.02 ‰ (n=5)* - *δ¹⁵N₍AIR₎: 15.06 ± 0.15 ‰ (n=5)* - *δ³⁴S₍VCTD₎: 17.23 ± 0.10 ‰ (n=4)* | ***B2215*** ***Run Date: 07/10/2021***   - *δ¹³C₍VPDB₎: –23.08 ± 0.10 ‰ (n=4)* - *δ¹⁵N₍AIR₎: 4.18 ± 0.06 ‰ (n=4)* - *δ³⁴S₍VCTD₎: 1.04 ± 0.23 ‰ (n=4)*   ***Run Date: 18/10/2021***   - *δ¹³C₍VPDB₎: –22.95 ± 0.12 ‰ (n=4)* - *δ¹⁵N₍AIR₎: 3.97 ± 0.11 ‰ (n=4)* - *δ³⁴S₍VCTD₎: 1.30 ± 0.36 ‰ (n=4)*   ***Run Date: 21/10/2021***   - *δ¹³C₍VPDB₎: –22.96 ± 0.14 ‰ (n=4)* - *δ¹⁵N₍AIR₎: 4.29 ± 0.05 ‰ (n=4)* - *δ³⁴S₍VCTD₎: 1.71 ± 0.20 ‰ (n=4)* |
| --- | --- |

*Modern Sample preparation*

*(i) Stable Isotope Analysis*

*Sample preparation:*

In the absence of scutes, the epidermis of softshell turtles is enveloped by a dense stratum of α-keratin [(Rodríguez et al. 2018)](https://paperpile.com/c/2eYXze/6mULr). To facilitate comparison with ancient collagen samples, it was imperative to access the collagen-rich dermal tissue [(Alibardi and Toni 2006)](https://paperpile.com/c/2eYXze/3qJUI) beneath. Consequently, we adhered to a standardised methodology employed for the preparation of sea turtle skin biopsies for stable isotope analysis [(Ceriani et al. 2014)](https://paperpile.com/c/2eYXze/hIPnh). This involved the separation of the epidermis from the underlying tissue, with an extra step of also separating the topmost keratinous layer. Subsequently, the separated skin biopsies underwent a thorough rinsing procedure with deionized water. The tissue was then subjected to freeze-drying and homogenization, after which it was weighed into tin capsules in preparation for stable isotope analysis.

*Stable Isotope Analysis*

Stable isotope analysis of the freeze-dried epidermis samples was conducted at the Marine Microbiology and Biogeochemistry stable isotope facility of the NIOZ Royal Netherlands Institute for Sea Research. Stable carbon (δ13C), nitrogen (δ15N), and sulphur (δ34S) isotopic compositions were determined using an Elementar Isoprime visION mass spectrometer continuous-flow isotope ratio mass spectrometer, which was interfaced with an Elementar Vario Isotope cube.

Samples underwent combustion in the presence of oxygen within a combustion reactor filled with tungsten oxide and a reduction reactor filled with copper wires, maintained at a temperature of 1150 °C and 850 °C, respectively. This process resulted in the production of nitrogen gas (N2), carbon dioxide (CO2), and sulphur dioxide (SO2). An EAS Wetsorber S filled trap was used to remove water generated during the combustion phase. Helium served as the carrier gas throughout the procedure. N_2_ gas was introduced directly into the mass spectrometer while CO_2_ and SO_2_ were collected on separate traps. After N_2_ was measured the CO_2_ trap was heated to release CO_2_ into the MS, after CO_2_ was measured the SO_2_ trap was heated to release SO_2_.

Certified acetanilide (Arndt Schimmelmann, Biogeochemical Laboratories Indiana University δ^13^C_VPDB_ = –29.53 ‰, δ^15^N_AIR_ = 1.18 ‰) was used a primary standard to normalise δ^13^C and δ^15^N values. IAEA-S2 and IAEA-S3, International Atomic Energy Agency (IAEA) (silver sulfide S2, δ^34^S_VCTD_ = 22.7 ‰; S3 δ^34^S_VCTD_ = –32.3 ‰) were used to normalise δ^34^S values. Results are reported as per mil (‰) relative to the internationally accepted standards VPDB, AIR and VCDT. Normalisation was checked using certified standard materials Casein (δ^13^C_VPDB_ = –26.98 ‰, δ^15^N_AIR_ = 5.94 ‰, and δ^34^S_VCTD_ = 6.32 ‰) and EMA-P1 (δ^13^C_VPDB_ = –27.85 ‰, δ^15^N_AIR_ = 0.055 ‰, and δ^34^S_VCTD_ = -3.01 ‰) from Elemental Micro Analysis, bovine liver (1577c; δ^13^C_VPDB_ = –17.55 ‰, δ^15^N_AIR_ = 8.06 ‰, and δ^34^S_VCTD_ = 2.54 ‰) from NIST and Sulfanilamide (δ^13^C_VPDB_ = –-28.2 ‰, δ^15^N_AIR_ = -2.56 ‰, and δ^34^S_VCTD_ = -9.57 ‰) from Elementar.

**Supporting Results**

***Table S1.*** *Curved carapace length (CCL), curved carapace width (CCW), weight (kg), and sex of the eight* Trionyx triunguis *sampled in Dalaman.*


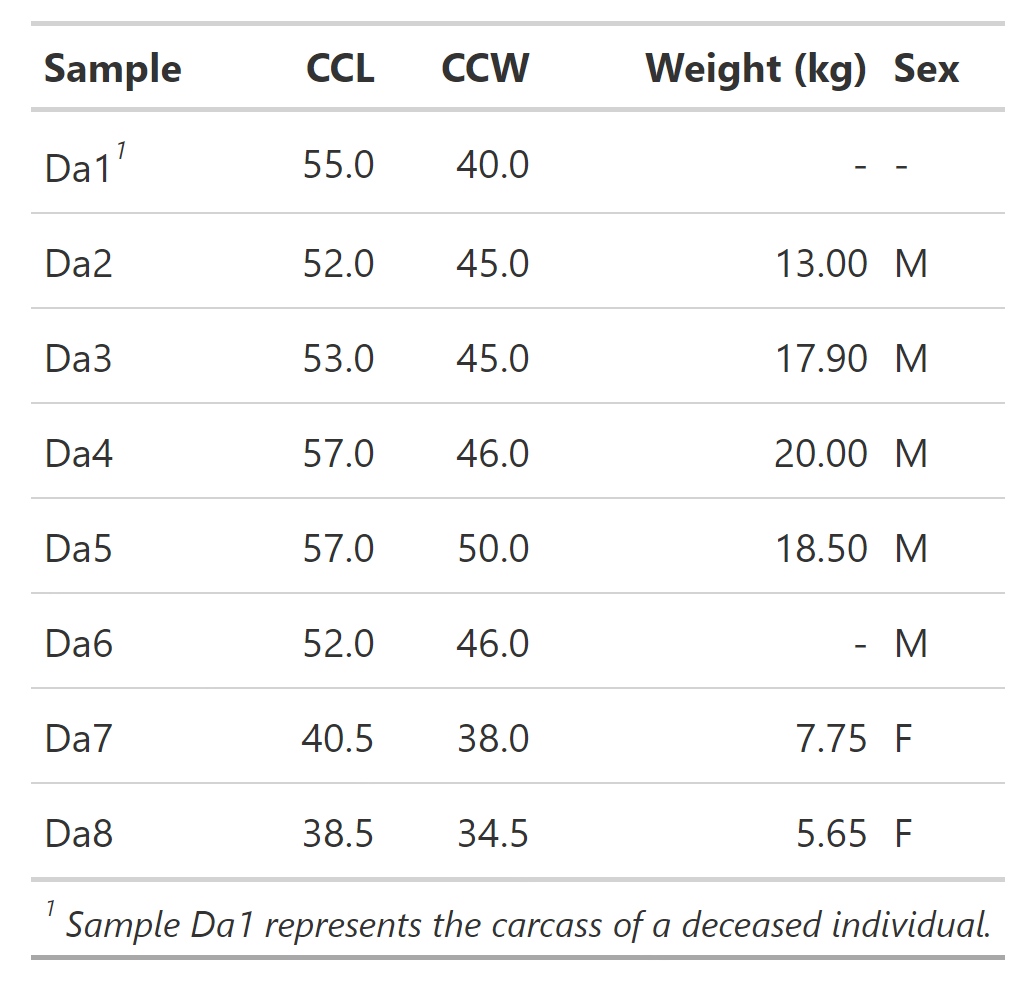


***Table S2.*** *Sample information and stable isotope measurements of modern and ancient* Trionyx triunguis *specimens. Quality control criteria which fell outside the range proposed for archaeological collagen* [*(DeNiro 1985; Nehlich and Richards 2009)*](https://paperpile.com/c/2eYXze/0PWix+yOUXI) *are displayed in red.*

*
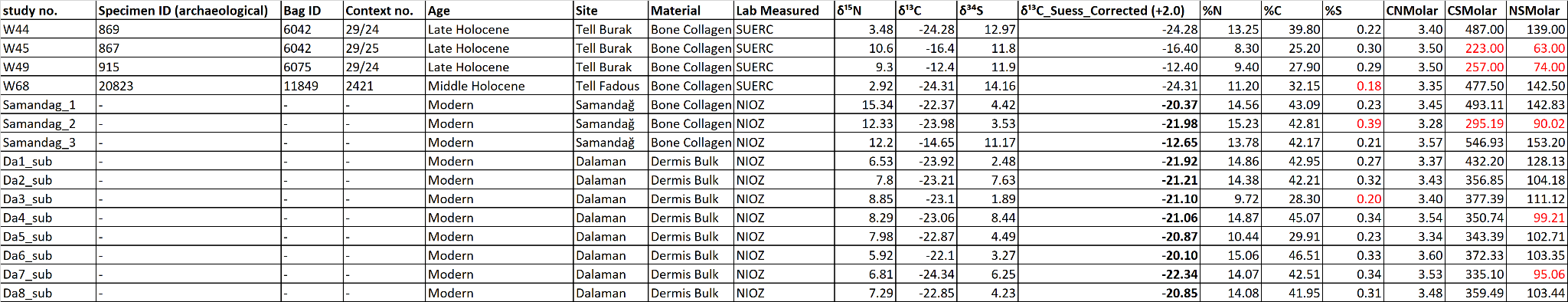
*

***Table S3.*** *Results of Pearson's product-moment correlation tests performed to evaluate the relationships between δ^34^S and three quality control (QC) criteria: the molar carbon to sulphur ratio (CS Molar), the molar nitrogen to sulphur ratio (NS Molar), and the percentage sulphur (%S). Provided are the t-statistic, degrees of freedom, p-value indicating significance, 95% confidence interval for the correlation coefficient, and the Pearson correlation coefficient.*


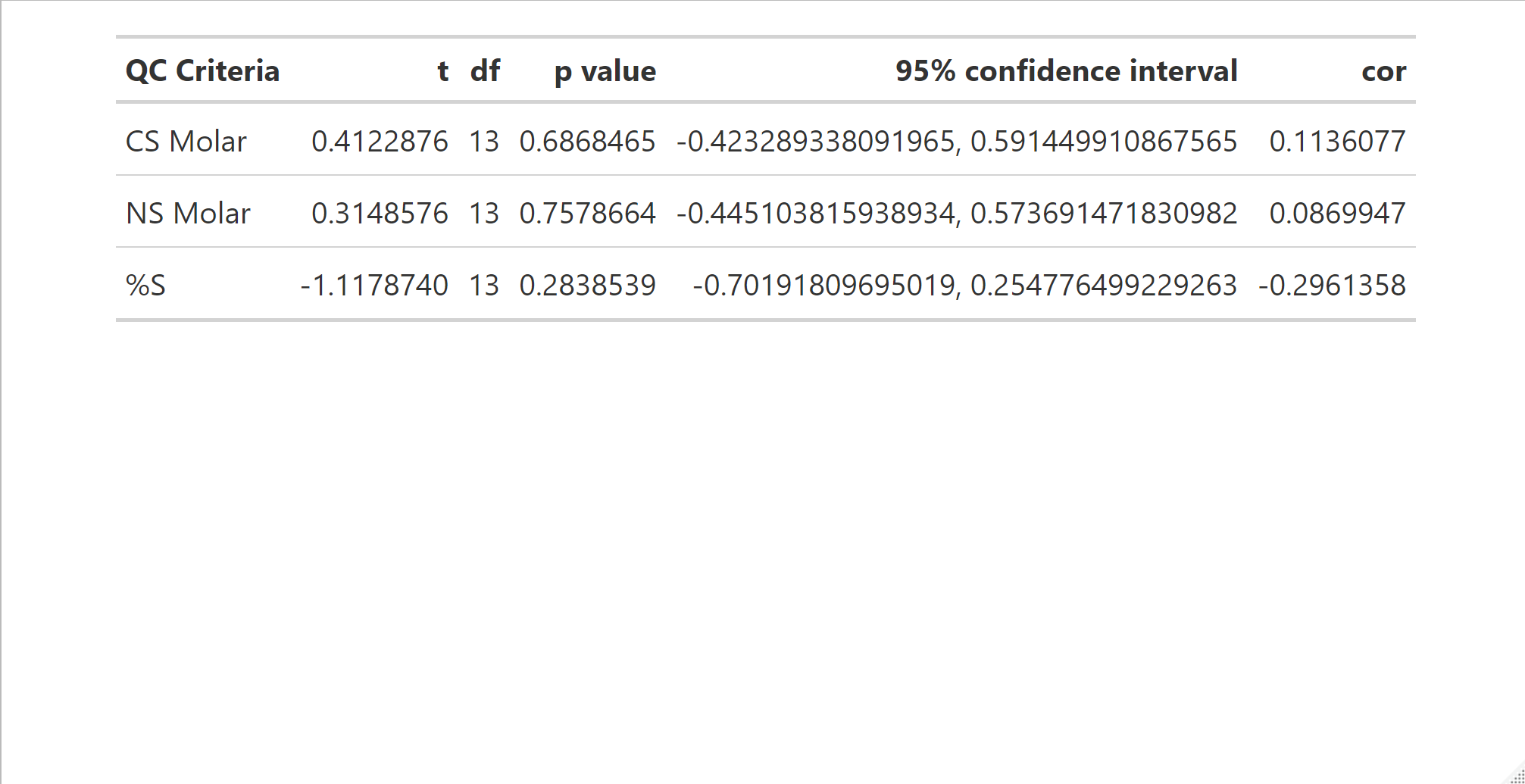


***Table S4.*** *Post hoc testing of pairwise comparisons of δ¹³C, δ¹⁵N, and δ³⁴S isotope values between sites (Samandağ, Dalaman, and Tell Burak). Differences between site pairs are reported alongside the 95% confidence intervals (CI) and associated p-values. Significant differences (p < 0.05) are in bold.*


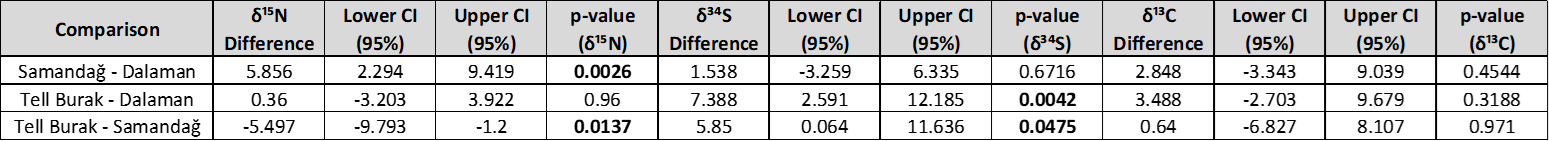


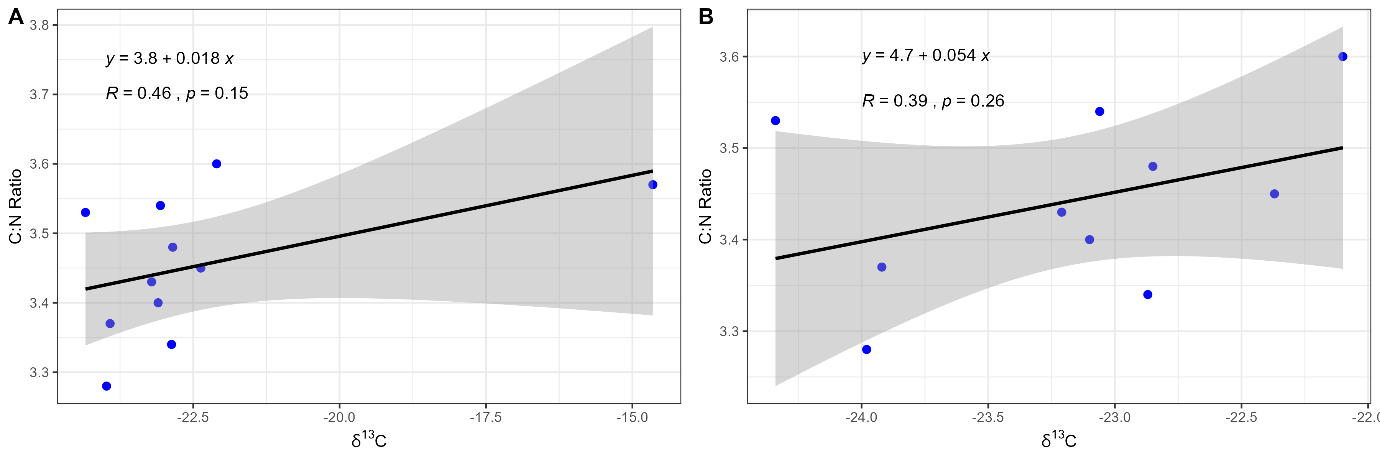


***Figure S1.*** *Pearson's product-moment correlation tests of δ¹³C vs C:N ratio in A: all modern* T. triunguis *skin samples, and B: all modern samples excluding Samandağ_3 which appears to have a separate ecological niche.*

***
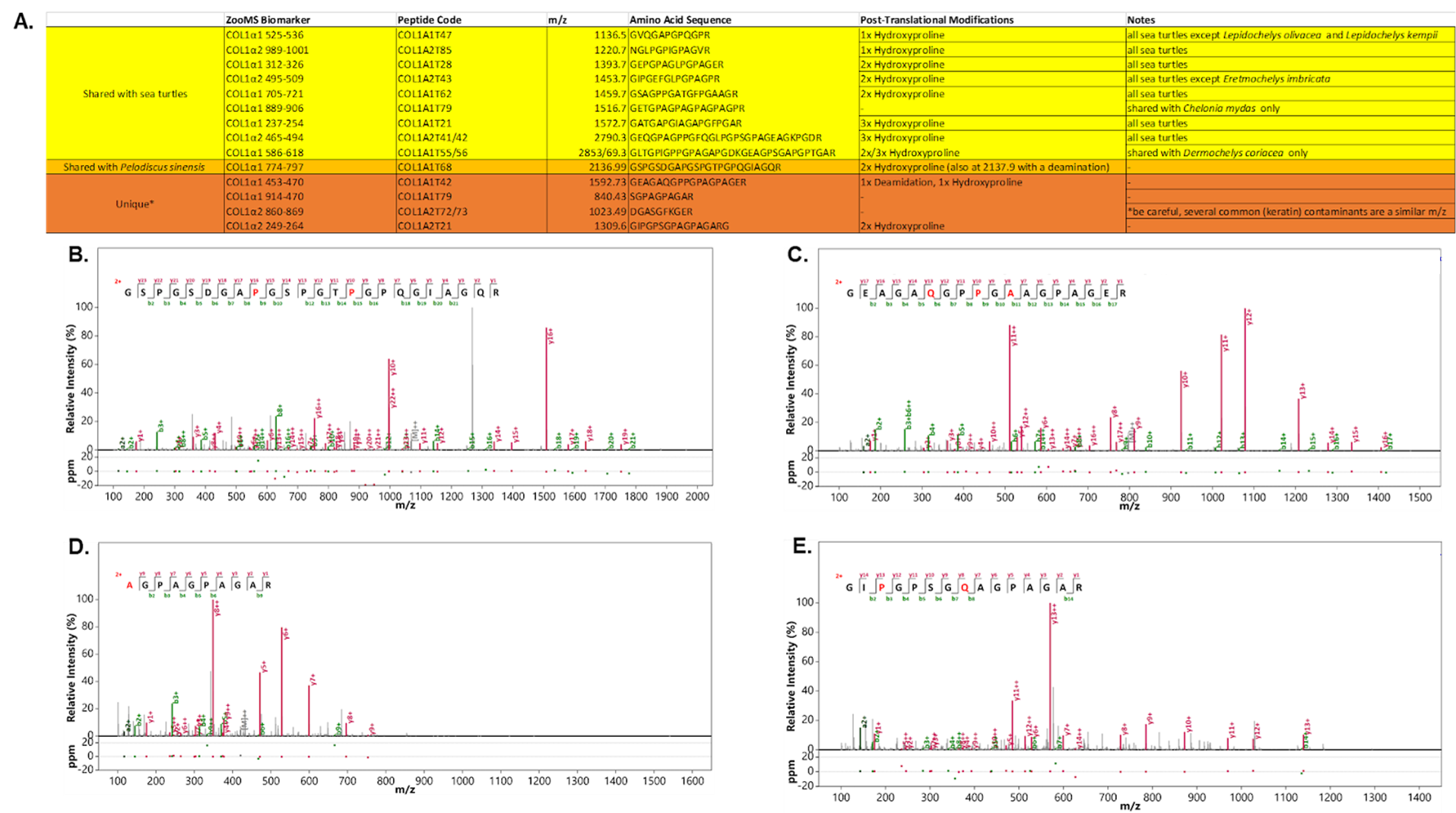
Panel S1.*** ***A)*** *Summary of ZooMS biomarkers identified in this study. Biomarkers are categorised as either shared with sea turtles* [*(Harvey et al. 2019)*](https://paperpile.com/c/2eYXze/jT3UK)*, shared with Pelodiscus sinensis (a softshell turtle native to China and Taiwan), or unique to T. triunguis. *In this context, "unique" refers to biomarkers that were not found in any of the tested turtle COL1 sequences.* ***(B-E)*** *LC-MS/MS spectra of the four reliable biomarkers presented in Figure 2 of the manuscript, visualised using pBuild. The spectra show coverage of Y and B ions, along with the amino acid substitutions and any post-translational modifications.*

**References**

[Alexander, Michelle M., Christopher M. Gerrard, Alejandra Gutiérrez, and Andrew R. Millard. 2015. “Diet, Society, and Economy in Late Medieval Spain: Stable Isotope Evidence from Muslims and Christians from Gandía, Valencia.” *American Journal of Physical Anthropology* 156 (2): 263–73.](http://paperpile.com/b/2eYXze/YWyMi)

[Alibardi, Lorenzo, and Mattia Toni. 2006. “Skin Structure and Cornification Proteins in the Soft-Shelled Turtle Trionyx Spiniferus.” *Zoology*  109 (3): 182–95.](http://paperpile.com/b/2eYXze/3qJUI)

[Buckley, Michael, Matthew Collins, Jane Thomas-Oates, and Julie C. Wilson. 2009. “Species Identification by Analysis of Bone Collagen Using Matrix-Assisted Laser Desorption/ionisation Time-of-Flight Mass Spectrometry.” *Rapid Communications in Mass Spectrometry: RCM* 23 (23): 3843–54.](http://paperpile.com/b/2eYXze/s7q5o)

[Çakırlar, Canan, Francis J. Koolstra, and Salima Ikram. 2021. “Tracking Turtles in the Past: Zooarchaeological Evidence for Human-Turtle Interactions in the Ancient Eastern Mediterranean.” *Antiquity* 95 (379): 125–41.](http://paperpile.com/b/2eYXze/R0hj)

[Ceriani, Simona A., James D. Roth, Llewellyn M. Ehrhart, Pedro F. Quintana-Ascencio, and John F. Weishampel. 2014. “Developing a Common Currency for Stable Isotope Analyses of Nesting Marine Turtles.” *Marine Biology* 161 (10): 2257–68.](http://paperpile.com/b/2eYXze/hIPnh)

[Cox, Jürgen, and Matthias Mann. 2008. “MaxQuant Enables High Peptide Identification Rates, Individualized P.p.b.-Range Mass Accuracies and Proteome-Wide Protein Quantification.” *Nature Biotechnology* 26 (12): 1367–72.](http://paperpile.com/b/2eYXze/Q7mei)

[DeNiro, Michael J. 1985. “Postmortem Preservation and Alteration of in Vivo Bone Collagen Isotope Ratios in Relation to Palaeodietary Reconstruction.” *Nature* 317 (6040): 806–9.](http://paperpile.com/b/2eYXze/0PWix)

[Harvey, Virginia L., Michelle J. LeFebvre, Susan D. deFrance, Casper Toftgaard, Konstantina Drosou, Andrew C. Kitchener, and Michael Buckley. 2019. “Preserved Collagen Reveals Species Identity in Archaeological Marine Turtle Bones from Caribbean and Florida Sites.” *Royal Society Open Science* 6 (10): 191137.](http://paperpile.com/b/2eYXze/jT3UK)

[Hermann Genz, Simone Riehl, Canan Çakırlar, Francesca Slim, and Alison Damick. 2016. “Economic and Political Organization of Early Bronze Age Coastal Communities: Tell Fadous-Kfarabida as a Case Study.” *Berytus* 55:79–119.](http://paperpile.com/b/2eYXze/vu3N)

[Höflmayer, Felix, Michael W. Dee, Hermann Genz, and Simone Riehl. 2014. “Radiocarbon Evidence for the Early Bronze Age Levant: The Site of Tell Fadous-Kfarabida (Lebanon) and the End of the Early Bronze III Period.” *Radiocarbon* 56 (2): 529–42.](http://paperpile.com/b/2eYXze/Fbxo)

[Janzen, Anneke, Kristine Korzow Richter, Ogeto Mwebi, Samantha Brown, Veronicah Onduso, Filia Gatwiri, Emmanuel Ndiema, et al. 2021. “Distinguishing African Bovids Using Zooarchaeology by Mass Spectrometry (ZooMS): New Peptide Markers and Insights into Iron Age Economies in Zambia.” *PloS One* 16 (5): e0251061.](http://paperpile.com/b/2eYXze/ngoBi)

[Kock, Willemien de, Meaghan Mackie, Max Ramsøe, Morten E. Allentoft, Annette C. Broderick, Julia C. Haywood, Brendan J. Godley, et al. 2023. “Threatened North African Seagrass Meadows Have Supported Green Turtle Populations for Millennia.” *Proceedings of the National Academy of Sciences of the United States of America* 120 (30): e2220747120.](http://paperpile.com/b/2eYXze/C9rk)

[Longin, R. 1971. “New Method of Collagen Extraction for Radiocarbon Dating.” *Nature* 230 (5291): 241–42.](http://paperpile.com/b/2eYXze/ny8et)

[Nehlich, Olaf, and Michael P. Richards. 2009. “Establishing Collagen Quality Criteria for Sulphur Isotope Analysis of Archaeological Bone Collagen.” *Archaeological and Anthropological Sciences* 1 (1): 59–75.](http://paperpile.com/b/2eYXze/yOUXI)

[Rodríguez, Carlos E., Ana María Henao Duque, Jennifer Steinberg, and Daniel B. Woodburn. 2018. “Chapter 34 - Chelonia.” *Pathology of Wildlife and Zoo Animals*, 825–54.](http://paperpile.com/b/2eYXze/6mULr)

[Sayle, Kerry L., Christopher R. Brodie, Gordon T. Cook, and W. Derek Hamilton. 2019. “Sequential Measurement of δ15 N, δ13 C and δ34 S Values in Archaeological Bone Collagen at the Scottish Universities Environmental Research Centre (SUERC): A New Analytical Frontier.” *Rapid Communications in Mass Spectrometry: RCM* 33 (15): 1258–66.](http://paperpile.com/b/2eYXze/rpsK4)

[Strohalm, Martin, Daniel Kavan, Petr Novák, Michael Volný, and Vladimír Havlícek. 2010. “mMass 3: A Cross-Platform Software Environment for Precise Analysis of Mass Spectrometric Data.” *Analytical Chemistry* 82 (11): 4648–51.](http://paperpile.com/b/2eYXze/ZDHiT)

[Vermeersch, Shyama, Britt M. Starkovich, Adriano Orsingher, and Jens Kamlah. 2022. “Subsistence Practices in Phoenicia and beyond: Faunal Investigations at Tell El-Burak, Lebanon (c. 725–350 BCE).” *Levant: The Journal of the Council for British Research in the Levant*, December, 1–19.](http://paperpile.com/b/2eYXze/NfKj)

[Wang, Le-Heng, De-Quan Li, Yan Fu, Hai-Peng Wang, Jing-Fen Zhang, Zuo-Fei Yuan, Rui-Xiang Sun, Rong Zeng, Si-Min He, and Wen Gao. 2007. “pFind 2.0: A Software Package for Peptide and Protein Identification via Tandem Mass Spectrometry.” *Rapid Communications in Mass Spectrometry: RCM* 21 (18): 2985–91.](http://paperpile.com/b/2eYXze/IJGAb)

[Winter, Rachel. 2023. “Marine Historical Ecology of Groupers (Epinephelidae) in the Eastern Mediterranean Utilising Zooarchaeology, Dietary Isotopes, Collagen Sequencing, and Peptide Mass Fingerprinting.” University of Groningen Press. https://doi.org/](http://paperpile.com/b/2eYXze/x1oQ)[10.33612/diss.680624364](http://dx.doi.org/10.33612/diss.680624364)[.](http://paperpile.com/b/2eYXze/x1oQ)

[Winter, Rachel M., Willemien de Kock, Meaghan Mackie, Max Ramsøe, Elena Desiderà, Matthew Collins, Paolo Guidetti, et al. 2023. “Grouping Groupers in the Mediterranean: Ecological Baselines Revealed by Ancient Proteins.” *Ecology and Evolution* 13 (10): e10625.](http://paperpile.com/b/SNLMku/DEN3)
