# Supplementary figures and images for: Stable isotope evidence of anthropocene disruption in African softshell turtle foraging
Source: PLoS One. 2026 Feb 11;21(2):e0339589. doi: 10.1371/journal.pone.0339589 (PMC12893573; doi:10.1371/journal.pone.0339589)

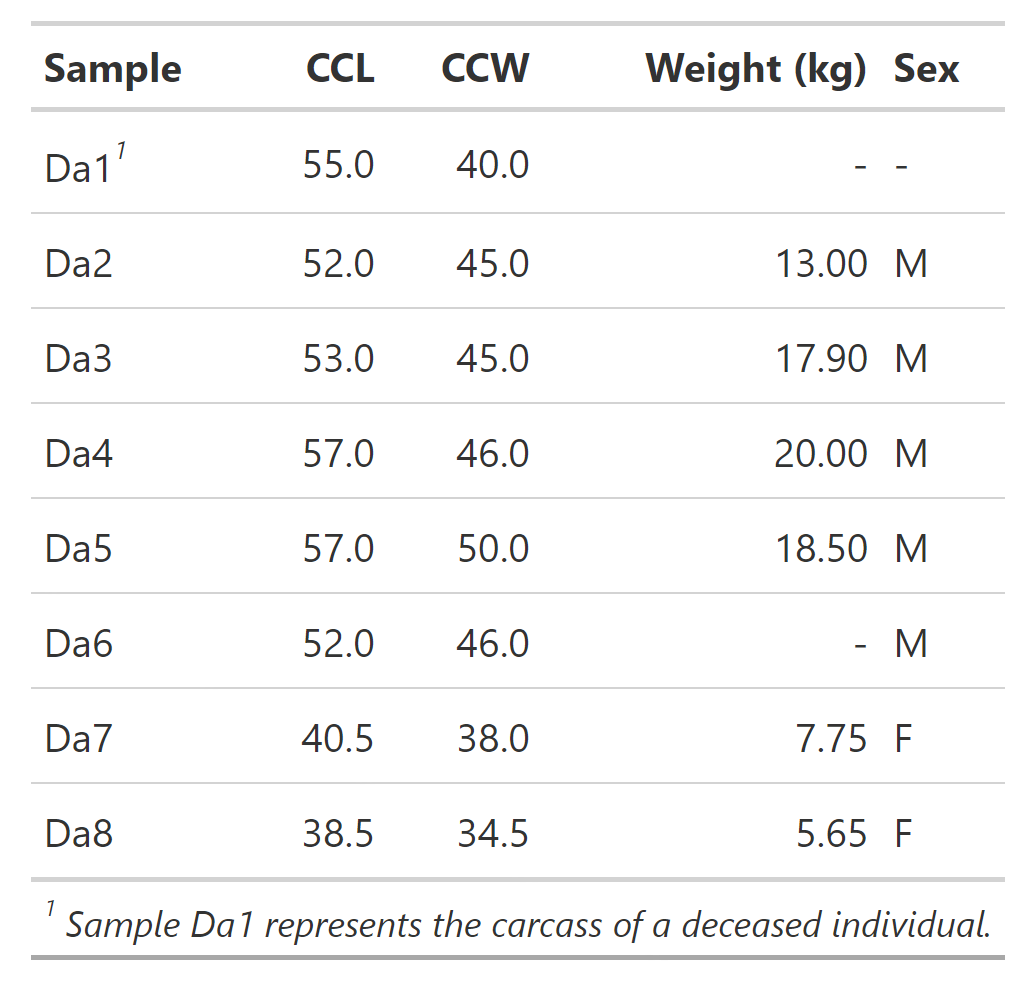

Supplement: S1 Table — (PNG) [file pone.0339589.s002.png]

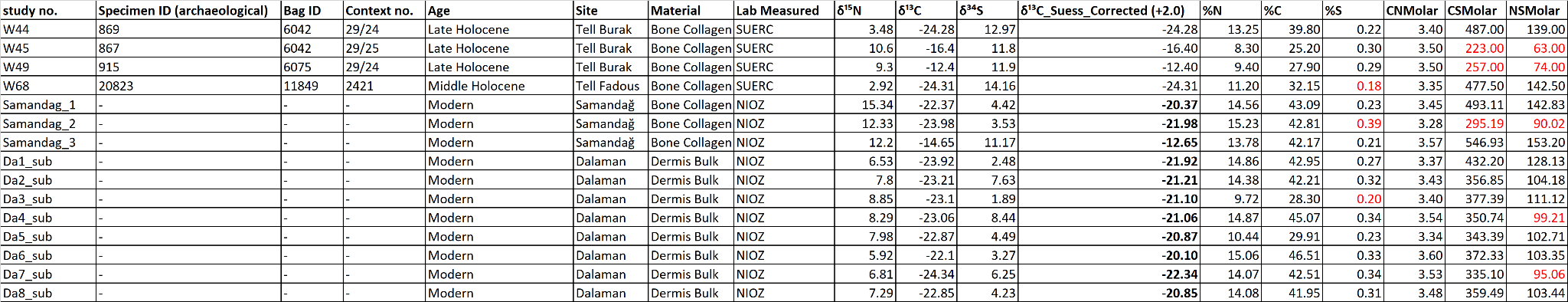

Supplement: S2 Table — Quality control criteria which fell outside the range proposed for archaeological collagen [30,31] are displayed in red. (PNG) [file pone.0339589.s003.png]

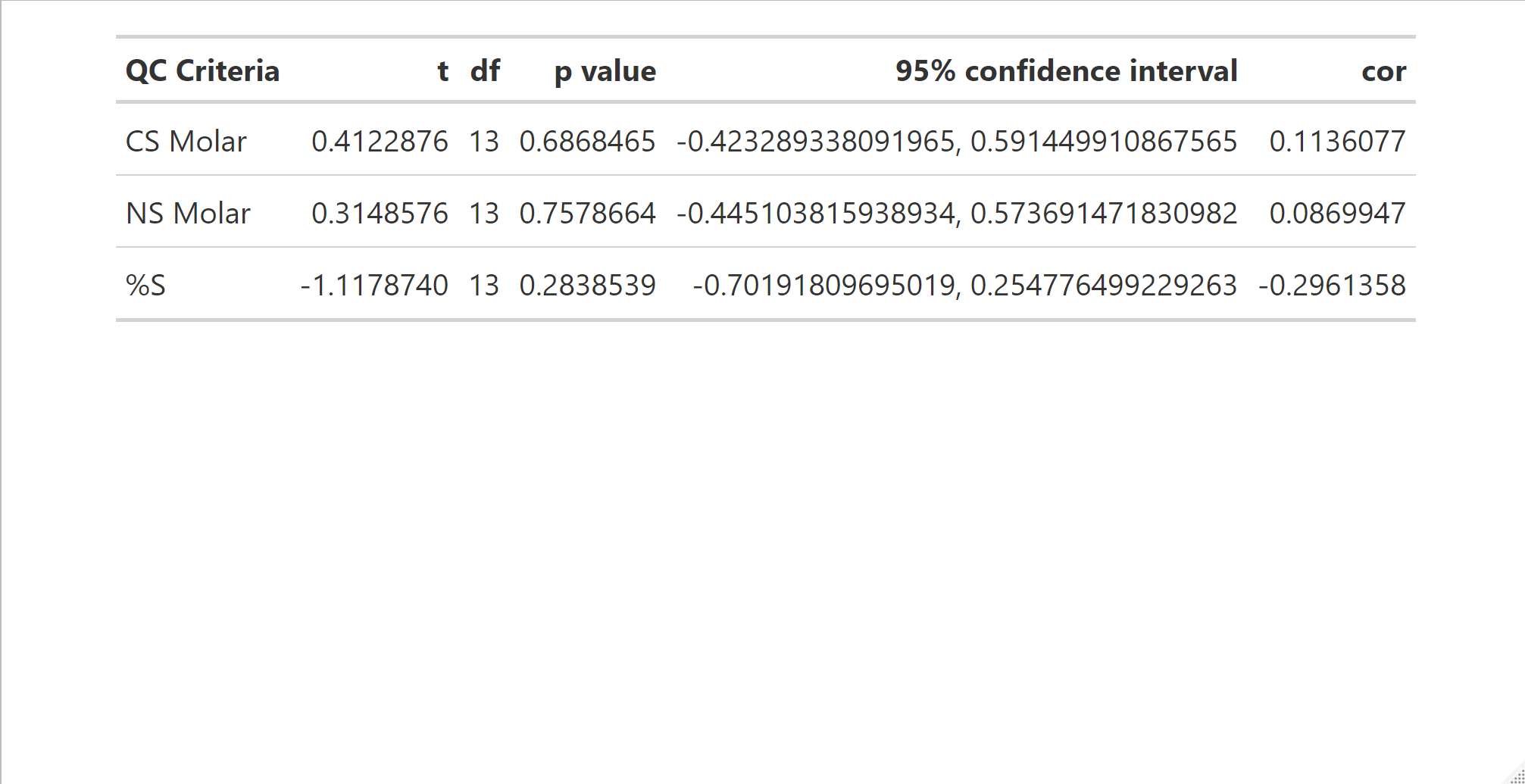

Supplement: S3 Table — Provided are the t-statistic, degrees of freedom, p-value indicating significance, 95% confidence interval for the correlation coefficient, and the Pearson correlation coefficient. (PNG) [file pone.0339589.s004.png]

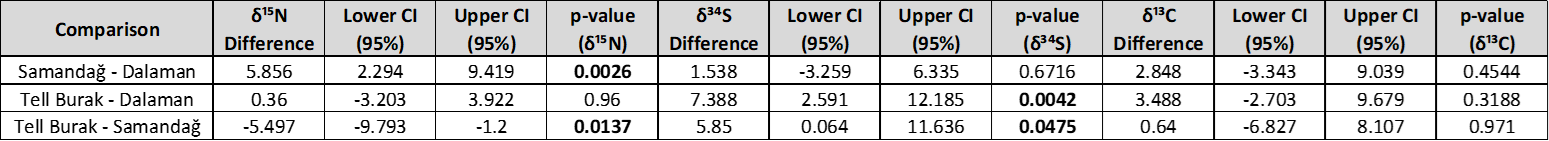

Supplement: S4 Table — Differences between site pairs are reported alongside the 95% confidence intervals (CI) and associated p-values. Significant differences (p < 0.05) are in bold. (PNG) [file pone.0339589.s005.png]

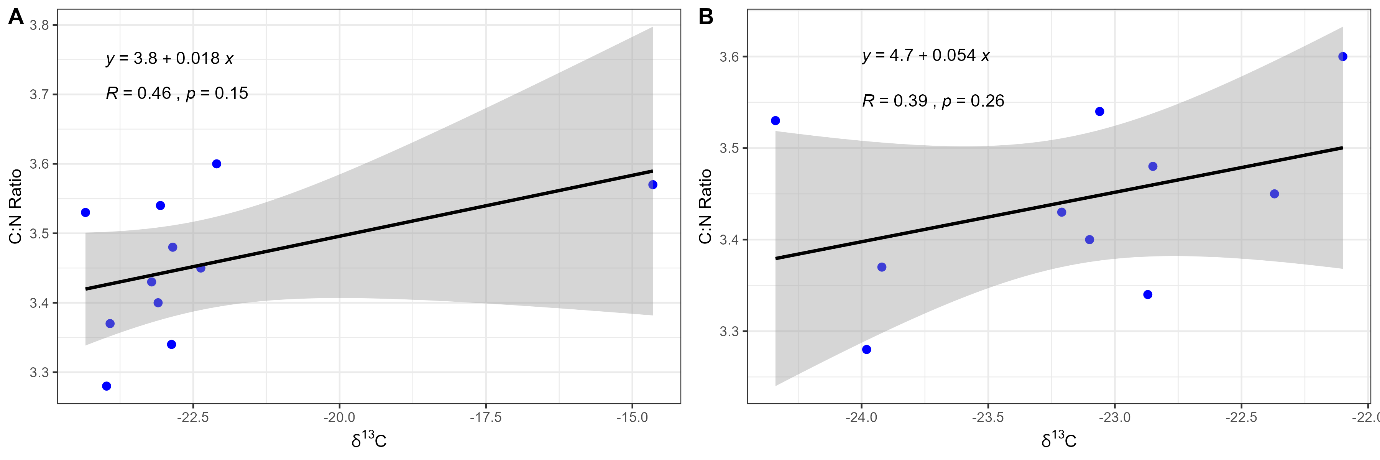

Supplement: S1 Fig — (PNG) [file pone.0339589.s006.png]

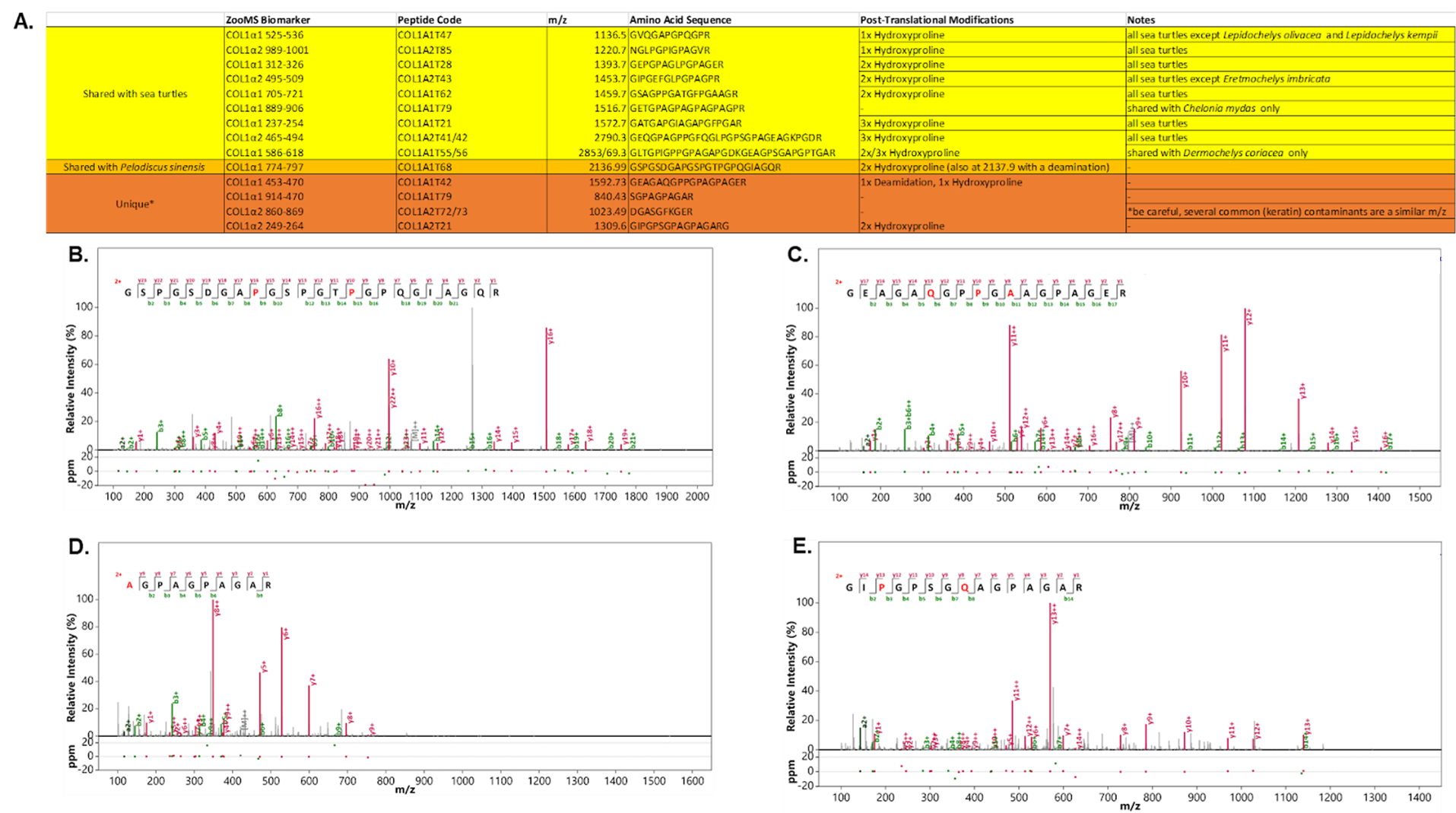

Supplement: S2 Fig — Biomarkers are categorised as either shared with sea turtles (Harvey et al. 2019), shared with Pelodiscus sinensis (a softshell turtle native to China and Taiwan), or unique to T. triunguis. *In this context, “unique” refers to biomarkers that were not found in any of the tested turtle COL1 sequences. (B-E) LC-MS/MS spectra of the four reliable biomarkers presented in Figure 2 of the manuscript, visualised using pBuild. The spectra show coverage of Y and B ions, along with the amino acid substitutions and any post-translational modifications. (PNG) [file pone.0339589.s007.png]
