# Supplementary material for: Stable isotope evidence of anthropocene disruption in African softshell turtle foraging
Source: PLoS One. 2026 Feb 11;21(2):e0339589. doi: 10.1371/journal.pone.0339589 (PMC12893573; doi:10.1371/journal.pone.0339589)
Supplement: Attachment — Submitted filename: Response to Reviewers.pdf [file pone.0339589.s009.pdf]

university of
groningen

12th November 2025

Dear Dr Paiva,

We sincerely thank you for the opportunity to revise our manuscript *Stable Isotope Evidence of Anthropocene Disruption in African Softshell Turtle Foraging* and for your detailed guidance. The majority of reviewer comments could be addressed directly, resulting in improvements to clarity, structure, and contextual framing.

While Reviewer 2 suggested the inclusion of additional ancient samples, this is unfortunately not feasible. African softshell turtle (*T. triunguis*) remains are extremely rare in the archaeological record; only a handful of specimens have been identified across countless bone fragments recovered from eastern Mediterranean coastal sites. In addition, access to Levantine archaeological material is highly restricted, and much of the region is currently inaccessible due to political, logistical, or legal constraints. Similarly, modern sampling of adult turtles is inherently challenging: the species is elusive, moves quickly to water when disturbed, and can only be sampled safely using specific trapping techniques at a limited number of river basins. Collectively, these factors make additional sampling not feasible.

Nevertheless, the current ancient and modern specimens are highly valuable. Despite their limited number, they provide rare and precious insights into Middle Holocene and contemporary African softshell turtle ecology, establishing *preliminary* "baselines" for dietary patterns and ecological signatures. When combined, the archaeological and modern data allow meaningful interdisciplinary comparisons, integrating zooarchaeology, stable isotope analysis, and palaeoproteomics (ZooMS). These comparisons provide novel insights (interpreted cautiously) into the ancient and contemporary ecology of this vulnerable and under-studied species, and contribute to a foundation for future research.

We have carefully addressed all other points raised by the reviewers and in your letter. Below we provide our detailed responses following the numbering of your letter. A clean version and a marked-up version of the revised manuscript are submitted alongside this document.

1. Manuscript style and naming

Thank you. The manuscript is in accordance with PLOS One style guidelines.

2. Updated Funding Statement

The funding statement has been amended as requested to include all relevant sources of support and the required final sentence:

> **Funding Statement:**
> The first author received support from the Marie Skłodowska-Curie Innovative Training Network *SeaChanges*, funded by the European Union's Horizon 2020 research and innovation programme (Marie Skłodowska-Curie grant agreement No. 813383), and from the Netherlands Sectorplan Social Sciences and Humanities (SSH, 2022). This study was funded by the Royal Netherlands Academy of Arts and Sciences (KNAW) Ecology Fund under grant number KNAWWF/747/ECO2021-17. There was no additional external funding received for this study.

3. Updated Acknowledgments

university of groningen

All funding-related information has been removed from the Acknowledgments section. The revised Acknowledgments now read:

Acknowledgments:
We gratefully acknowledge Dr. Simona Ceriani for her valuable advice on skin biopsy processing, and M. Munoz-Alegre for her mass-spectrometry support. Our sincere thanks go to Dr. Bektaş Sönmez for providing the Samandağ specimens. Skin biopsies were exported to the University of Groningen under CITES permit 22NL305802/11. Archaeological samples are stored in the zooarchaeological collection of the Groningen Institute of Archaeology (University of Groningen). Our research would not have been possible without the work of the Tell Fadous-Kfarabida and Tell el-Burak excavation teams. Many thanks to S. Ikram. We also appreciate D. Klingberg Johansson and the National History Museum of Denmark for permitting us to sample *T. triunguis* for proteomic analysis.

4. Data availability

All underlying data will be made openly available at the time of publication. We confirm full compliance with PLOS ONE's data sharing policy.

5. Blot and gel data

Not applicable. No blot or gel images are included in this manuscript.

6. Ethics statement

The full ethics statement has been added to the Methods section. It reads:

> "This study was conducted in accordance with the regulations of the Directorate of Nature Conservation and National Parks of the Ministry of Agriculture and Forestry, Turkey, which granted research permission for the fieldwork. Ethical approval for sampling procedures was obtained from the Animal Experiments Ethics Committee of Aydın Adnan Menderes University (Permit Number: 64583101/2021/039). The biopsy location on each turtle was selected to minimize disturbance, and all handling was performed using clean gloves. Turtles were processed as quickly and gently as possible to reduce stress and were released immediately into their natural environment after sampling, within 5 minutes. No turtles were sacrificed in this study."

7. Copyrighted map (Figure 1)

Thank you for noting this. Figure 1 has been replaced with a version that avoids any copyright conflicts. The updated figure and caption now comply fully with the CC BY 4.0 license.

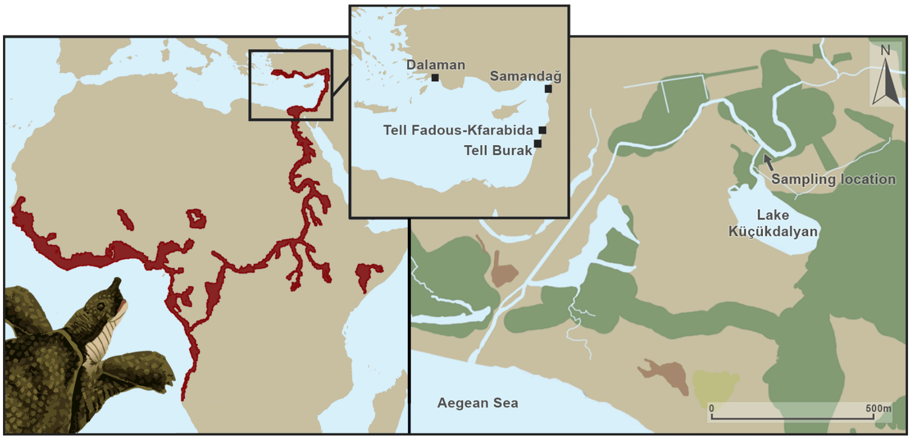

8. Supporting Information captions

Captions for all Supporting Information files have been added to the end of the manuscript.

Response to Reviewers

We sincerely thank both reviewers for their constructive comments and suggestions, which have helped us to improve the clarity and quality of the manuscript. Our responses are provided below, with reviewer comments in *italics* and our responses following each in plain text.

Reviewer #1

This manuscript presents an isotope study of modern and ancient (Middle Holocene) softshell turtle remains in the Eastern Mediterranean. [...] I support its publication. I have a few recommendations, detailed below, which can be easily dealt with.

We thank the reviewer for their supportive and insightful comments. We have addressed each point below.

Sample size is small and should be mentioned in the abstract. Ancient turtles (n=4) exhibit more diverse diets...

We appreciate this comment. Sample size is often an issue when dealing with archaeological remains, and it is not our intention to obscure this limitation. Line 54 in the abstract now reads: "Ancient turtles (n=4) from the Levant (modern day Lebanon) exhibit more diverse diets.." and lines 140-143 in the Introduction now reads "In this study, we explore these questions through a small but informative dataset (ancient n=4, modern n=11), aiming to identify whether stable isotope values from both modern and archaeological *T. triunguis* specimens show evidence of marine foraging and to what extent such signatures are consistent between time periods and regions.", explicitly noting sample size and justification earlier on.

Archaeological samples come from the Lebanese coast, not Turkey.

Thank you. Line 54 in the abstract now reads "Ancient turtles (n=4) from the Levant (modern day Lebanon) exhibit more diverse diets..", clearly indicating that the archaeological material

derives from the Lebanese coast.

Line 105: phrasing is confusing; Line 139: figure reference incorrect.

Thank you, these errors have been corrected. Line 108 now reads “The oldest published Holocene specimen..” and Line 160 now points to Fig. 1, not Fig. 2.

Methods: bold font unnecessary and distracting.

We agree. The bold formatting was originally added to highlight information required by the journal office. It has now been removed.

Reviewer #2

In general, this is a well-structured and interesting paper. For the first time, the authors use stable isotope and ZooMS analysis to explore dietary shifts in modern and ancient African softshell turtles. The novelty of this research is clear, and the new ZooMS markers are valuable additions to the field. The authors have also tried to make their data as accessible as possible, while clarifying several limitations of their research.

My main concern regarding this research is that the limited data presented in the paper cannot answer the three questions raised in the introduction.

- *Whether their foraging behaviours favour marine or freshwater environments?*

With only eight modern samples and four ancient samples, it is impossible to answer this question.

- *How these behaviours may have changed over time?*

Again, with only eight modern samples, three Iron Age samples and one Bronze Age sample, it is impossible to answer this question. It makes no sense to discuss temporal changes.

- *If this behaviour is universal or specific to certain populations*

I did not see the authors discuss this point at all.

Therefore, I ask the authors to add more ancient samples, although I don’t know if any are available. With the current limited dataset, the pattern is unclear to me. I would not recommend accepting this paper since the argument cannot be supported by solid evidence. However, under the current framework, it would be a high-quality paper if more data were added.

We thank the reviewer for their thoughtful and constructive comments. We fully acknowledge that the sample size, particularly of the ancient specimens, is limited. Unfortunately, additional archaeological *T. triunguis* remains are not currently accessible. The species is rare in the archaeological record, and access to material from much of the Levant is presently restricted. This makes the specimens included in our study especially valuable.

Furthermore, the modern isotope dataset and the newly identified ZooMS markers independently represent significant contributions. We have revised the manuscript to make these limitations explicit and clearly state that our ancient results are *preliminary*. Observed

patterns are presented cautiously, emphasizing marine foraging and population-level variation without claiming definitive temporal or behavioural trends.

Revised text excerpts:

> **Lines 95–98:** Their presence as bycatch in the eastern Mediterranean Sea (Taskavak, Reimann, and Polder 1999; Türkozan, Ilgaz, and Yılmaz 2006) suggests that some individuals opportunistically forage in marine settings. Whether such marine foraging behaviour can be detected across time or populations, however, remains uncertain.
>
> **Lines 99–107:** There have been no studies addressing whether modern and ancient populations of *T. triunguis* show similar patterns of environmental use or dietary signatures. Any comparison of past and present remains therefore provides an opportunity to investigate potential continuity or divergence in foraging strategies. Moreover, it is not yet clear whether the observed ecological plasticity of *T. triunguis* is a universal trait of the species or one that emerges only in certain local populations. Addressing these uncertainties can provide insight into the functional roles of *T. triunguis* within their ecosystems (Lovich et al. 2018), which may inform conservation and management strategies of this understudied species.
>
> **Lines 140–143:** In this study, we explore these questions through a small but informative dataset (ancient n=4, modern n=11), aiming to identify whether stable isotope values from both modern and archaeological *T. triunguis* specimens show evidence of marine foraging and to what extent such signatures are consistent between time periods and regions.
>
> **Lines 148–154:** Next, we compared stable isotope values (δ13C, δ15N, and δ34S) from newly identified prehistoric bones and freshly sampled modern individuals to assess broad-scale patterns rather than definitive temporal trends. This study represents the first stable isotope and historical ecology analysis of *T. triunguis*, offering preliminary insights into its past and present ecological roles and testing the potential of isotopic data to inform future studies on ecological plasticity in the African softshell turtle.

Other comments:

1. *Explain choice of C, N, and S isotopes.*

A paragraph has been added to the Introduction (Lines 127-139).

2. *Introduce ecological and environmental context.*

Expanded in the Supplementary Information. Line 185–186 now reads: “Expanded descriptions of the archaeological sites are present in the supplementary information.”

3. *Are there any differences between Tell el-Burak and Tell Burak?*

No, there are not. All references standardized to *Tell el-Burak*.

4. *Differentiate sexes in Figures 3B–D.*

Done.

5. *Figure 2 difficult to read.*

The 300 dpi PNG file version has improved legibility.

Closing Remarks

We thank the editor and both reviewers again for their time and constructive feedback. The manuscript has been carefully revised in response to all comments and has been substantially improved.

Sincerely,

Dr. Willemien de Kock
on behalf of all co-authors
University of Groningen
w.de.kock@rug.nl
